# Psychological Effect of Discrete Outbreak Events of COVID-19 on Health Information Search in China

**DOI:** 10.3390/bs13020109

**Published:** 2023-01-28

**Authors:** Yan Liu, Peng Zou

**Affiliations:** School of Management, Harbin Institute of Technology, Harbin 150001, China

**Keywords:** discrete outbreak events, health information search, COVID-19, geographical distance

## Abstract

Community transmission events occasionally happened in the long-term pandemic, which led to repeated outbreaks of COVID-19. In addition to potential physical threats, the outbreaks could also lead to psychological stress and influence their health behaviors, especially for vulnerable people. It poses a great challenge to both physical and mental health management. However, little is known about the impacts of discrete outbreak events of COVID-19 on people’s reactions to health concerns in the long-term pandemic period. In the current study, we discussed the impact of discrete outbreak events of COVID-19 on health information search for specific symptoms in China from a perceptive of susceptibility. The empirical study was conducted after the first wave of outbreak events ended in China from June to October 2020. Three typical outbreak events happened during this period, and a total of 1800 search index data for 60 cities in China crawled from Baidu search engine were included in the data set. Using the real-world searching data, we conducted a panel data analysis to examine the psychological effect of discrete outbreak events on the symptom search and the moderation effect of the geographical distance. It was found that discrete outbreak events significantly increased the symptom search, and its impacts were various in different regions. More health information search caused by discrete outbreak events was found in cities which were closer to the outbreak area. Implications for healthcare were discussed.

## 1. Introduction

The COVID-19 pandemic has dramatically impacted people’s life worldwide, since its outbreak in late December 2019 in Wuhan, China. There had been more than 662 million confirmed cases, and over 6.7 million deaths had been reported to WHO as of early January 2023 [1]. Moreover, the situation may be worse, because the death cases only explained the respiratory distress preceding deaths and who were linked to infections of COVID-19. According to the estimates from the WHO, there were approximately 14.9 million excess deaths associated directly or indirectly with the COVID-19 pandemic from January 2020 to December 2021, with only 5.42 million reported COVID-19 deaths [2,3]. The pandemic has led to considerable economic and societal disruptions and adverse psychological consequences [4,5]. To contain the virus outbreak, prevention measures have been carried out in many countries, including lockdowns, mask-wearing orders, transport restrictions, vaccination, and wide quarantine [6,7]. However, community transmissions of COVID-19 still happen occasionally due to its characteristics of being highly contagious and mutated [8].

Different from the first wave of the outbreak which was particular salient to people, discrete outbreak events were supposed to change people’s health behavior in an imperceptible way in the longer pandemic time. As people’s response to discrete community transmissions was not as hysterical as to the first lapse of the outbreak, its impact was likely to be ignored or received less attention. However, contagious cues (e.g., community transmissions) would trigger a host of instinctive reactions (e.g., emotional response and safety-seeking behaviors), especially for the population with similar identifies (e.g., experiencing typical symptoms), regardless of whether they are facing a real risk of harm (e.g., no infection cases in their cities) [9,10]. According to previous studies, perceived similarity could increase their risk perception of specific threat cues [11]. For instance, perceived similarity to women who contract a disease (e.g., breast cancer) would increase people’s perceived susceptibility beyond its medical risk [12]. In the context of COVID-19, people who were experiencing respiratory symptoms were expected to perceive greater similarity to infected persons, as they all could be identified as susceptible populations.

Specifically, we supposed that occasional COVID-19 outbreak events could raise stronger responses for people with suspicious symptoms (e.g., cough and fever) due to their perceived susceptibility. It was likely for them to suffer from unnecessary worries about infection risks and associated consequences (such as economic cost, behavioral restrictions, and social isolation) so as to influence their mental health (e.g., anxiety) [13] and subsequent health behaviors (e.g., health information search, physical examinations, and doctor visits) [14]. In addition to that, more health behaviors would also be performed to promote physical and mental recovery, such as exercise rehabilitation and natural environment interaction [15,16]. More natural and human factors would be involved in healthcare during the long-term COVID-19 [17]. That would pose more challenges to healthcare management. However, little is known about how discrete outbreak events will impact people’s health-related behaviors, especially for the health information search, which is always considered as an indicator of the tendency of future behaviors.

Health information search is a prevalent behavior for people to cope with their health concerns [18,19,20,21]. According to an industry report in 2013, about 59% of the US adults used the Internet to search for health information [19]. In addition, the percentage of Europeans is about 55%, depending on a survey conducted in 2020 [20]. Especially during the global health crisis, online health information search would be more common, as people tried to avoid face-to-face consultation and turn to the Internet [22,23]. For example, a recent study found a 30% increase in health-related search in Italy due to COVID-19 [24].

The most common motivation for health information-seeking behavior is to learn about a health concern and provide information support for self-diagnosis [19,25,26]. People would actively search for health information to cope with health concerns, which is helpful for them to alleviate uncertainty, reduce anxiety, prepare for doctor’s appointments and support decision-making [27,28,29,30]. In particular, online health information search is an easily accessible way for people to satisfy their needs for healthcare during the global pandemic. Hence, we believed people would search for health information to deal with their escalated concerns caused by discrete outbreak events.

Based on the preceding discussion, we supposed that discrete community transmissions events of COVID-19 could increase health information search referring to the symptoms of common respiratory diseases. Furthermore, we expected the effect could vary across geographical regions due to different psychological distances.

Psychological distance describes how individuals perceive distance to certain events [31]. In the context of health risks, the psychological distance has been found to influence people’s emotions, risk perceptions, and behaviors [32,33,34]. For example, Yang and McAllister (2020) found feeling psychologically close to the measles outbreak could lead to intense anxiety and fear, higher risk perception, and a stronger willingness for information engagement and vaccination [32]. In addition, So and Nabi (2013) found a reduced psychological distance to sexually transmitted diseases (e.g., HIV/AIDS) would increase their perceived personal risk (e.g., feeling more vulnerable to getting diseases) [35]. In summary, these studies show that psychological proximity is associated with high perceived susceptibility and strong reactions to risks. If people perceive the risk event as psychologically close to them, they are more prone to considering it as dangerous and take action. In our study, we supposed that the influence of discrete outbreak events of COVID-19 would be amplified with psychological proximity. We particularly focused on the geographical distance to the outbreak area, a well-known dimension for the evaluation of psychological distance [36]. People would perceive closer to the event if they lived closer to the area where the event happened. Thus, we expected people who lived closer to the outbreak city could have a stronger response (e.g., health information search) to their respiratory symptoms when facing COVID-19 local outbreak events, and vice versa.

Based on the discussions above, we proposed our hypotheses as following:

**H1:**  *Discrete community transmission events of COVID-19 would increase people’s health information search.*

**H2:**  *The impact of discrete outbreak events on health information search could be larger for cities which were closer to the outbreak area relatively to distant ones. The geographical distance from target cities to the infectious region negatively moderates the effect of discrete community transmission events of COVID-19 on health information search.*

The purpose of this study is to explore how discrete outbreak events of COVID-19 will impact people’s health information searching behavior. It is important to have a deeper insight into health information search, as it could be useful to support health management and avoid potential hazards.

Firstly, searching data provides us with a good opportunity to capture people’s intentions for healthcare. For example, some research has used searching data for symptom keywords to predict people’s mental health, needs for healthcare, and future health behaviors [21,37,38,39]. That would be useful for health organizations to monitor people’s health state and provide healthcare support.

Secondly, providing attention to health information search is helpful to avoid its potential harm. Even though online health information seeking brings us major potential benefits, it sometimes leads to unexpected costs [26,40]. For example, White and Horvitz (2009) found that web search engines have the potential for people to escalate their health concerns about common symptoms [41]. In addition, Vismara et al. (2021) found that health information seeking and cyberchondria (heightened attention to serious medical concerns based on the searching information on the Web) would lead to higher psychological stress during the COVID-19 pandemic [24]. The escalation of health concerns would contribute to a higher level of anxiety, excessive search, and occupations of medical resources (e.g., frequent doctor visits, repeated examinations, and unnecessary treatments) [14,42,43]. Furthermore, medical experts have also argued for action to prevent unnecessary health anxiety triggered by health information [44].

Consequently, it is important to investigate whether and how discrete outbreak events of COVID-19 will impact people’s health information search. The results could contribute to the allocation of healthcare resources and provide mental healthcare support during the long-term pandemic period.

We conducted a panel data analysis to test our hypotheses using the real-world searching data and made a pilot survey to investigate the health information-searching behavior in China to discuss the practical significance of our study. We expected the results could assist in formulating healthcare policies and optimizing resources allocation.

## 2. Data and Method

### 2.1. Data

#### 2.1.1. Health Information Search

Many studies have used search data to measure people’s health state and effects, such as predicting influenza epidemics and depression [21,37]. Following the previous literature, we used search data to measure people’s psychological stress and medical demands. We collected the daily-city-level search data for typical symptoms on Baidu, the most popular search engine in China. We crawled the data of the search indices of typical symptoms keywords (cough, sore throat, etc.) for 60 cities in China from June 2020 to October 2020. The data covered the provincial capitals and the top 50 cities in GDP.

Because COVID-19 is a kind of respiratory disease, we supposed that people who were experiencing common symptoms of respiratory diseases (e.g., cold/flu and rhinitis) could have a higher level of anxiety about their health issues, resulting in more health information search. Hence, we collected the search data using the keywords for the common symptoms in Table 1. Then, we aggregated the search index data of the listed keywords to construct the dependent variable.

#### 2.1.2. Discrete Community Transmission Events of COVID-19

Wuhan’s lockdown was ended in early April 2020, which signaled a successful process for Chinese people to return to normal life. However, community transmissions still happened after 56 days without increments of local infections. Since the lockdown lifted, three typical community transmission events happened during the period we concerned (Beijing, 13 June 2020; Qingdao, 11 October 2020; and Kashi 24 October 2020). To test our hypothesis, we obtained the search data before and after each event happened and used a dummy variable to indicate whether an outbreak event happened on that day. To rule out the effect of seasonal factors on health information search (e.g., seasonal variation of incidence of respiratory diseases), we collected the search data for five days before and after the event happened, depending on a reasonable assumption that there was no significant fluctuation in incidence within a short period window. Finally, we had a total of 1800 observations in our panel data set.

We also did a post-check for history infection cases data during the period of our data and found no transmission risks out of the outbreak cities due to the strict prevention policy in China. That could rule out the potential noises of the physical effect of COVID-19 and provide an additional proof for the psychological effect of outbreak events.

#### 2.1.3. Geographical Distance

**Figure 1:**
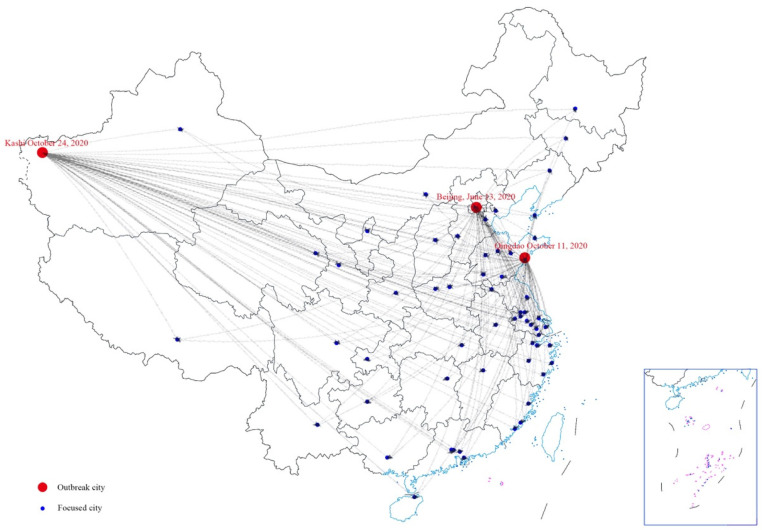
Sketch map for the psychological spillover effect of outbreak events.

We measured the geographical distance as a moderator variable. We calculated the geographical distance from each city to the outbreak city using the data of the latitude and the longitude which were obtained through the Amap open platform API. A sketch map for the psychological spillover effect of outbreak events is shown in Figure 1.

#### 2.1.4. Baidu Search

People’s searching behavior could also be influenced by other factors (e.g., breaking news). For example, breaking news may lead to higher internet usage and then increase people’s health information search due to convenience. Hence, we collected the search data with the keywords of “Baidu” to measure the general Internet usage of the Baidu search engine as a control.

### 2.2. Method

In this study, we conducted an empirical analysis in China during the long-term COVID-19 period. The panel data set was constructed with 60 cities in China from June 2020 to October 2020.

To test the effect of discrete community transmission events of COVID-19 on health information search and the moderation effect of the geographical distance, we conducted a fixed effect panel data analysis and proposed the model as following:

$$\begin{array}{c} \mathrm{Symptoms}\_{\mathrm{Search}}_{\mathrm{it}}\\ \;=\beta_{0}+\beta_{1}\mathrm{COVID}\_19\_{\mathrm{Event}}_{t}+\beta_{2}{\mathrm{Distance}}_{\mathrm{it}}+\beta_{3}\mathrm{COVID}\_19\_{\mathrm{Event}}_{t}{*\mathrm{Distance}}_{\mathrm{it}}\\ \;+\beta_{4}\mathrm{Baidu}\_{\mathrm{search}}_{\mathrm{it}}+\beta_{5}{\mathrm{Month}}_{t}+\overset{N-1}{\underset{n=1}{\sum }}\beta_{6n}{\mathrm{City}}_{i}+\mu_{\mathrm{it}} \end{array}$$

where ${\mathrm{Symptoms}\;\mathrm{Search}}_{\mathrm{it}}$ is the symptoms search index of city i at the day t, $\mathrm{COVID}\_19\_{\mathrm{Event}}_{t}$ is a dummy variable to measure whether the outbreak events happen on day t, and ${\mathrm{Distance}}_{\mathrm{it}}$ is the geographical distance from city i to the outbreak city, specifically, to Beijing, Qingdao, and Kashi depending on day t in which event period window. We also included the $\mathrm{Baidu}\_{\mathrm{search}}_{\mathrm{it}}$ to measure the Internet usage which may affect searching behavior as a control. The city- and month-fixed effects are used to account for the city heterogeneity and the seasonal effect. $\beta_{1}$ and $\beta_{3}$ are our coefficients of interest which capture the main effect of discrete community transmission events of COVID-19 and the moderation effect of the geographical distance. To adjust the non-normal distribution and impact of the excessive magnitude difference, we normalized the search index and the geographical distance by performing a log transformation.

## 3. Results

The descriptive statistics and correlations are provided in Table 2.

Based on the model mentioned before, the coefficient of COVID-19 events estimates the main effect of discrete outbreak events of COVID-19 on health information search. If the coefficient is significantly positive, hypothesis 1 would be verified. $\beta_{3}$ is the other coefficient of interest for our study. According to our hypothesis, the closer the geographical distance is, the stronger the impact of outbreak events on health information search would be. That also means the effect would be weakened along with the increment of the distance. Thus, if the coefficient of the interaction term is significantly negative, hypothesis 2 would be verified.

We estimated the fixed effect regression model with the robust standard error to account for the potential autocorrelation and heteroscedasticity. The main results are presented in Table 3.

Model 1 showed a significant positive effect of discrete community transmission events of COVID-19 on health information search ($\beta_{1}$ = 0.015, *p* < 0.05). That indicated the health information search significantly increased after outbreak events happened. Thus, hypothesis 1 was verified. Based on model 1, the distance and the two-way interaction term of the distance and outbreak events were gradually added into model 2 and model 3. The coefficient of $\mathrm{COVID}\_19\_{\mathrm{Event}}_{t}{*\mathrm{Distance}}_{\mathrm{it}}$ was significantly negative ($\beta_{3}$ = −0.030, *p* < 0.01). The results showed that the impact of outbreak events on health information search would vary across different cities and decreased along with the distance to the COVID-19 outbreak city. Namely, people who lived closer to the outbreak city would have more health information search, when the discrete community transmission event happened. Thus, hypothesis 2 was verified. Our results showed that the respiratory-related symptoms search was significantly increased after the community transmission events happened, but the increment varied across geographical regions.

To further support our results, we conducted a survey referring to health information search through an online questionnaire platform—Wenjuanxing (https://www.wjx.cn/) (accessed on 1 January 2023) in July 2020 in China. It was helpful for us to verify the validity of using health information to indicate people’s psychological state and healthcare needs and emphasize its importance to healthcare management.

We mainly investigated the frequency, motivations, and functions of health information searching behavior in China. A total of 291 valid questionnaires were collected with a valid rate of 89.26% by adopting a snowball strategy. The details of the demographic characteristics are presented in Table 4.

For the frequency of health information search behavior, participants were asked to indicate how often they search for health-related information (1 = never, 5 = often). The results show that 96.91% of the subjects used the Internet to search for health information, and 59.8% did this frequently (i.e., responses were sometimes and often) (see Table 5). That means a relatively high percentage of people would like to search for health information online.

For the motivations of health information search, we asked them to choose possible reasons for their health information search when experiencing physical complaints (Table 6). “Self-diagnosis” (75.26%) is the most important reason, followed by “eliminate the uncertainty about health issues” (72.85%). “Relieve worries or anxiety about health problems” and “Help them make decisions about the health issues” are also common reasons with response rates of 69.07% and 65.98%, respectively. There were 4.81% of participants indicating other motivations, such as “understand the symptoms” and “seek the guidance of treatment”. This provides an additional proof that people usually use health information search to support their health management in China, which could serve as a valid indicator of needs for healthcare and psychological support.

For the functions of health information search, we asked participants to indicate how much they agreed or disagreed with the statements about their health information search experience referring to the functions (1 = completely disagree, 5 = strongly agree). To simplify our results, we categorized the results into agree (strongly agree and relatively agree), disagree (completely disagree and relatively disagree), and neutral (Table 7). Same as the discussions before, the effect of health information search was ambiguous with a relatively high percentage of participants choosing the neutral options (neither agree nor disagree). In addition, a total of 42.27% of the participants stated health information search could ease their health concerns or anxiety; however, 43.30% thought it sometimes exacerbated their health concerns or anxiety. Furthermore, 42.61% of participants believed the search behavior could help them get a sense of security, but 16.15% disagreed and 41.24% held neutrality. In addition, only 35.05% of participants agreed the health problem was not as serious as previously thought after searching health information online, which indicated a relatively low effectiveness of anxiety relieving. The statistics show that, even though we hope the health information search could release people’s uncertainty and anxiety about their health concerns, its performance may be not always as expected. Moreover, it could exacerbate people’s anxiety and raise unnecessary fear about imagined serious consequences sometimes.

In summary, the survey showed that health information search was common in people’s daily life which reflected people’s psychological stress and healthcare needs. Even though it was expected to alleviate health anxiety, it could also bring potential harm. 

## 4. Discussion

Our results suggested that discrete outbreak events of COVID-19 would stimulate health information searching behavior for people who experienced typical symptoms. A reasonable explanation for this phenomenon may be fears about the consequences of being infected, such as health harm, economic cost, and social isolation. Especially for people with suspicious identities (e.g., cough symptom), they may be eager to search for health information to eliminate their uncertainty about health concerns when receiving news about COVID-19 outbreak events. Our results also found the search increment was various across geographical regions. We thought that could be a reason of different evaluations of the psychological distance to infection risks. If people lived closer to the outbreak city, they might have stronger emotional responses (such as fear and anxiety), perceive a higher level of risk and consequently search for more health information online. Our findings revealed the impact of discrete outbreak events of COVID-19 on health information search during the long-pandemic period in China.

Health information search was expected to relieve people’s psychological stress; however, it sometimes led to a higher level of anxiety. Especially for people who have little or no medical training, searching online would increase their health anxiety and even escalate their medical concerns [41]. It called for more understanding and attention to health information searching behavior. Our results indicated that the discrete outbreak event of COVID-19 was a factor that would increase people’s health information search. It could be helpful for healthcare agencies to predict people’s medical needs and avoid potential psychological problems.

### 4.1. Study Strengths

Our study enriched the literature referring to health information search and COVID-related topics. Firstly, we introduced discrete outbreak events in the long-term period as an influential factor in health behavior. The prior studies mostly focused on the impact of COVID-19 on people’s behaviors in the first wave [45,46]; however, less attention has been paid to discrete outbreak events in the long-term period of COVID-19. This study extended the literature by demonstrating the psychological effect of discrete outbreak events of COVID-19 on health information search. 

Secondly, we specifically focused on the symptoms searching behavior in daily life. Previous studies have shown that the pandemic increased people’s Internet search about the topic of COVID-19 generally, such as coronavirus, protective behavior, vaccine and immunity, emotions, and panic buying [22,47]. Different from the existing research, we focused on the impact of discrete outbreak events on a common behavior in daily life, specifically the symptom-searching behavior in the long pandemic period. We found that discrete outbreak events could increase people’s search for typical symptoms. This showed outbreak events could lead to a highly enhanced demand for healthcare of people who experience respiratory-related symptoms. This indicated the vulnerable population would likely perceive higher psychological stress when outbreak events happen. In addition, we proposed that the geographical distance would affect the impact of discrete outbreak events on health information search. We addressed the geographical distance as a relevant characteristic when considering the heterogeneity influence of outbreak events in different cities.

Furthermore, this study extended the literature referring to COVID-19 and psychological stress. Prior research has studied psychological stress in the context of COVID-19 [22,24]; for instance, Pourhaji et al. (2022) have examined the effect of perceived threat of COVID-19 and individual characteristics on stress responses [48]. However, limited studies considered people’s response to common health issues as an outcome of the psychological consequences of COVID-19. To further understand the influence of psychological stress on health behavior, this study tried to figure out how health information search will be affected in the face of COVID-19. In addition, we introduced the regulatory role of the geographical distance on risk perception to explore the heterogeneity of psychological effects in different environments. 

Our findings also have practical implications. First, our results confirmed that in the long period of the pandemic, discrete outbreak events would still cause health anxiety or escalate health concerns resulting in more health information search. This provides suggestions for the government and health organizations to pay attention to people’s mental health and medical needs in addition to control measures for the epidemic. Second, the results showed that people in cities that were closer to the outbreak area would experience higher anxiety and call for more healthcare support. This would help the government to better adjust its medical resources across different regions. Furthermore, both the results of our survey and previous studies figured out that health information search might also lead to psychological stress (e.g., excessive anxiety). This may cause unnecessary health behaviors (e.g., persistent reassurance seeking, frequent doctor visits, expensive examinations, and necessary treatments) which are costly for both consumers and the society [14,42,43]. We suggested policymakers provide interventions, for example, refusing rumors, popularizing health knowledge, and providing psychological support to reduce people’s excessive anxiety and make people have reasonably cognition about their health problems.

### 4.2. Limitations and Implication for Future Research

Our research has several limitations. First, our study was limited to the context of China. Due to different epidemic prevention policies, people’s attitudes and adaptability to COVID-19 could vary in different countries. Further studies can test the impact of discrete community transmission events or virus mutation events on people’s reactions under different backgrounds. Second, we analyzed people’s health information searching behavior at the city level. Although the search data can suitably reflect people’s actual reactions, we could not obtain individual characteristics due to the data limitations. Future research can test how individual characteristics will influence their health behaviors when facing discrete outbreak events. Third, there could be some limitations of using data mining to demonstrate the causal relationship. Even though we tried to reduce the interference to our results by adopting several measures (such as using a short period window in data analysis, checking history infection cases as a control, and examining the moderation effect of the geographical distance), noises of confusing factors could not be completely avoided in the real-world context. Experiments studies are expected in future research to explain the mechanism of the psychological effect of COVID-19 on the subject.

## 5. Conclusions

This study aimed to investigate people’s reactions to discrete community transmission events during the long period of the COVID-19 pandemic in China. Through the panel regression analysis, we found the positive impact of discrete outbreak events on people’s health information search about typical symptoms of respiratory diseases. In addition, the geographical distance would moderate the relationship between outbreak events and health information search. This study demonstrated different reactions across geographical regions. This study may encourage further research on psychological consequences in the long-term period pandemic in China and make some suggestions for healthcare management.

## Figures and Tables

**Table 1:** Keywords for typical symptoms.

Categories	Keywords
Airway disorders	Cough, dry cough, wet cough, excessive phlegm, wheezing, and short of breath
Nasal symptoms	Stuffy nose, itchy nose, runny nose, and sneeze
Throat symptoms	Dry throat, sore throat, and itchy throat
Others symptoms	Fever and headache

**Table 2:** Descriptive statistics and correlations.

Variables	Mean	SD	Min	Max	(1)	(2)	(3)	(4)	(5)
(1) Symptom_Search (ln)	6.841	0.550	4.060	8.057	1				
(2) Baidu_Search (ln)	8.609	0.878	6.736	10.85	0.910 ***	1			
(3) COVID_19_Events	0.500	0.500	0	1	0.028	0.016	1		
(4) Distance (ln)	7.136	1.162	0	8.368	0.010	−0.045 *	0.000	1	
(5) Month	0.667	0.472	0	1	0.085 ***	0.018	0.000	0.252 ***	1

Notes: *n* = 1800; *** *p* < 0.01; * *p* < 0.1.

**Table 3:** Estimation results of the effects of discrete community transmission events of COVID-19 on health information search.

Variable	(1)	(2)	(3)
COVID_19_Events	0.015 **	0.015 **	0.230 ***
	(0.007)	(0.007)	(0.044)
Distance		−0.004	0.011 **
		(0.004)	(0.005)
COVID_19_Events * Distance			−0.030 ***
			(0.006)
Baidu_search	0.573 ***	0.570 ***	0.574 ***
	(0.044)	(0.044)	(0.044)
Month	0.080 ***	0.083 ***	0.083 ***
	(0.008)	(0.008)	(0.008)
Constant	1.844 ***	1.904 ***	1.759 ***
	(0.376)	(0.379)	(0.378)
Observations	1800	1800	1800
R-squared	0.171	0.172	0.183
Number of cities	60	60	60
City Fixed Effects	YES	YES	YES

Notes: Robust standard errors in parentheses; *** *p* < 0.01; ** *p* < 0.05; * *p* < 0.1.

**Table 4:** Demographic details of respondents (*n* = 291).

Parameter		*n* (%)
Gender	Male	115 (39.52%)
	Female	176 (60.48%)
Age (in years)	18 to 25	67 (23.02%)
	26 to 30	71 (24.40%)
	31 to 40	96 (32.99%)
	41 to 50	37 (12.71%)
	51 or older	20 (6.87%)
Education background	Junior high school and below	6 (2.06%)
	High school/technical secondary school	9 (6.53%)
	Junior college	46 (15.81%)
	Undergraduate	142(48.80%)
	Postgraduate or above	78 (26.80%)
Annual disposable income level	Below 30,000	76 (26.12%)
	30,000 to less than 60,000	74 (25.43%)
	60,000 to less than 100,000	51 (17.53%)
	100,000 to less than 200,000	55 (18.90%)
	Above 200,000	35 (12.03%)
Resident city distribution	Northeast China	86 (29.55%)
	East China	139 (47.77%)
	West China	46 (15.81%)
	Middle China	20 (6.87%)

**Table 5:** Frequency of health-related search.

	Frequency	Percent
Never	9	3.1%
Rarely	25	8.6%
Occasionally	83	28.5%
Sometimes	84	28.9%
Often	90	30.9%
Total	291	100%

**Table 6:** Motivations for health information search.

Item	Frequency	Percent
Help me with my self-diagnosis	219	75.26%
Help me eliminate the uncertainty about my health	212	72.85%
Relieve my worries or anxiety about health problems	201	69.07%
Help me make decisions about the health issues	192	65.98%
Other	14	4.81%

**Table 7:** Functions of health information search.

Item	Agree *n* (%)	Disagree *n* (%)	Neutral *n* (%)
Health information search could ease my health concerns or anxiety	123 (42.27%)	56 (19.24%)	112 (38.49%)
Health information search sometimes exacerbates my health concerns or anxiety	126 (43.30%)	57 (19.59%)	108 (37.11%)
In most cases, searching for health information help me gain the sense of security	124 (42.61%)	47 (16.15%)	120 (41.24%)
In most cases, I feel the health problems are not as serious as previously thought after searching health information online	102 (35.05%)	60 (20.62%)	129 (44.33%)

## Data Availability

Data can be obtained from the corresponding author based upon request.
